# Study protocol: analysis of regional lung health policies and stakeholders in Africa

**DOI:** 10.1186/s12961-020-00618-5

**Published:** 2020-12-09

**Authors:** Claire Jensen, Emma Heneine, Brenda Mungai, Violet Murunga, Hleziwe Hara, Rose Oronje, Angela Obasi, Bertie Squire, Eliya Zulu, On behalf of The IMPALA Consortium, Bertie Squire, Kevin Mortimer, Angela Obasi, Rachel Tolhurst, Miriam Taegtmeyer, Jahangir Khan, Louis Niessen, Imelda Bates, Bertrand Mbatchou, Amsalu Binegdie, Emmanuel Addo-Yobo, Eliya Zulu, Hellen Meme, Hastings Banda, Jamie Rylance, Adegoke Falade, Heather Zar, Lindsay Zurba, Brian Allwood, Maia Lesosky, Asma El Sony, Nyanda Elias Ntinginya, Beatrice Mutayoba, William Worodria

**Affiliations:** 1 African Institute for Development Policy (AFIDEP), Petroda Glasshouse, Area 14 – plot number 14/191, P.O. Box 31024, Lilongwe, Malawi; 2 Liverpool School of Tropical Medicine (LSTM), Liverpool, United Kingdom

**Keywords:** Lung health policy, policy stakeholder analysis, Africa lung health, tuberculosis, non-communicable diseases

## Abstract

**Background:**

Lung health is a critical area for research in sub-Saharan Africa. The International Multidisciplinary Programme to Address Lung Health and TB in Africa (IMPALA) is a collaborative programme that seeks to fill evidence gaps to address high-burden lung health issues in Africa. In order to generate demand for and facilitate use of IMPALA research by policy-makers and other decision-makers at the regional level, an analysis of regional lung health policies and stakeholders will be undertaken to inform a programmatic strategy for policy engagement.

**Methods and analysis:**

This analysis will be conducted in three phases. The first phase will be a rapid desk review of regional lung health policies and stakeholders that seeks to understand the regional lung health policy landscape, which issues are prioritised in existing regional policy, key regional actors, and opportunities for engagement with key stakeholders. The second phase will be a rapid desk review of the scientific literature, expanding on the work in the first phase by looking at the external factors that influence regional lung health policy, the ways in which regional bodies influence policy at the national level, investments in lung health, structures for discussion and advocacy, and the role of evidence at the regional level. The third phase will involve a survey of IMPALA partners and researchers as well as interviews with key regional stakeholders to further shed light on regional policies, including policy priorities and gaps, policy implementation status and challenges, stakeholders, and platforms for engagement and promoting uptake of evidence.

**Discussion:**

Health policy analysis provides insights into power dynamics and the political nature of the prioritisation of health issues, which are often overlooked. In order to ensure the uptake of new knowledge and evidence generated by IMPALA, it is important to consider these complex factors.

## Background: lung health in Africa

Globally, it is estimated that 1 billion people, the majority of whom live in low- and middle-income countries, suffer from acute and chronic respiratory conditions [1, 2]. The five principal conditions that contribute to the global burden of respiratory diseases are asthma, chronic obstructive pulmonary disease, acute respiratory infections, tuberculosis (TB) and lung cancer [1]. The estimated burden in sub-Saharan Africa (SSA) is large and growing. For example, lower respiratory tract infections are the single biggest cause of mortality in SSA, killing almost 1 million Africans each year [3], and the WHO Africa region accounts for one-quarter (25%) of global TB cases and deaths [4, 5]. However, despite the high morbidity and mortality associated with many lung diseases, they are still likely to be “*under-estimated, under-diagnosed, under-treated as well as inadequately prevented*” [6]. There is an urgent need for lung health research in SSA to address this evidence gap and support the design and implementation of evidence-informed prevention, diagnosis and treatment interventions.

### The International Multidisciplinary Programme to Address Lung Health and TB in Africa

The International Multidisciplinary Programme to Address Lung Health and TB in Africa (IMPALA) is a 4-year collaborative programme led by the Liverpool School of Tropical Medicine and funded by the United Kingdom National Institute of Health Research that seeks to fill evidence gaps to address high-burden lung health issues in Africa, many of which lack sufficient funding and research evidence. IMPALA comprises 22 research and advocacy institutions, including partners in 10 African countries, that has been intentionally constituted to span the WHO Africa Region. It engages in multi-disciplinary collaborative research across clinical and public health, applied social science, health systems, health economics and modelling, policy, and implementation science disciplines to produce implementable solutions to critical issues surrounding lung health. The work focuses specifically on intersections between non-communicable lung disease, acute lung disease, air pollution and tobacco-related disease as well as how each of these interact with TB.

IMPALA’s Pathways to Impact component has the overarching goal of creating demand for and enabling the use of evidence generated through IMPALA in lung health policy decisions, programmes and practice. Among other activities, this will be achieved by identifying the policy factors influencing the behaviours and environments that protect or harm lung health through policy and stakeholder analyses at the regional level. This analysis of policies and stakeholders will be inclusive of the five regions of the African Union, including North Africa. The regional analyses will then inform the development and implementation of a comprehensive policy engagement and evidence uptake strategy for IMPALA. The strategy will be a living document that will be informed by three phases of analysis and adapted as necessary throughout the life of the programme.

## Methodology and analysis

The study will be conducted in three phases as depicted diagrammatically below.

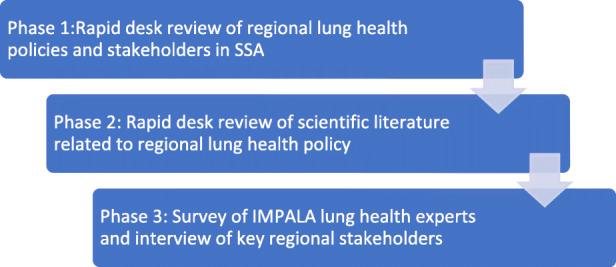



### Phase 1: rapid desk review of regional lung health policies and stakeholders in SSA

This first phase of work seeks to answer the following questions:

1. What regional policies exist to tackle lung health challenges in the region?
2. What lung health issues are prioritised in these policies? What lung health issues are not prioritised in these policies?
3. Who are the main actors in lung health policy development and advocacy at the regional level (including donors)?
  a What are their interests and roles?
  b Where is there potential for IMPALA to expand key actors’ involvement in lung health/TB?
4. What opportunities exist for regional policy engagement with key stakeholders?

#### Methodology

This analysis will draw from a rapid desk review of key stakeholders in SSA as well as their lung health/TB policies, programmes and platforms. Platforms eligible for inclusion should have content on decision-making, policy, experts, leaders, heads of state, ministries of health, ministers, stakeholder engagement and evidence dissemination. Relevant content will be added to the shared Excel sheet.

Lung health and/or TB-specific organisations will be identified through Google searches using the following terms [Lung OR TB OR tuberculosis OR respiratory] AND [chronic OR acute] AND [strategy OR policy OR plan OR framework OR program* OR advoca* OR organi* OR network OR fund] AND [Africa] AND [East* OR South* OR West* OR Central]. Websites of the identified eligible organisations and of regional economic, governance and public health bodies (e.g. African Union, East Africa Community, Southern African Development Community, Africa CDC) will be scanned to find policy and programme documents/information related to TB/lung health. Documents/information included in the review and analysis will be those describing acute or chronic lung health policies, strategies, frameworks, programmes and advocacy efforts. Documents/information not focusing on lung health policies, plans, strategies, frameworks, programmes and their implementation and evaluation, and decision-making/advocacy platforms will be excluded from the review. The organisations with relevant TB/lung health policies and/or programmes will be further reviewed to identify platforms for TB/lung health-related policy engagement. In particular, reviewers will search the relevant organisations’ websites and google with the following terms [conference OR technical working group OR TWG OR forum OR meeting OR policy OR practice] AND [organisation name OR organisation abbreviation]. The following content will be extracted by two reviewers using an Excel sheet shared between the reviewers: policy/programme/platform name, date and aim; stakeholders involved, roles, interests [developer(s), funder(s), implementer(s), target population]; policy options, interventions or activities; gaps and implementation challenges, including factors influencing policy/programme design and implementation; and role of evidence.

#### Analysis

After data collection, four researchers will discuss the content in the shared Excel sheet to reach consensus on themes and trends in (1) lung health policies or strategies at regional level; (2) lung health issue(s) prioritised; (3) integration of lung health diseases with other diseases/health issues; (4) key regional actors in lung health policy development and advocacy; (5) structures for discussing and advocating for lung health policy; (6) factors that influence lung health policies in Africa; (7) investment in lung health programme implementation; and (8) the role of research/evidence in lung health policy development, implementation and advocacy.

### Phase 2: rapid qualitative desk review of scientific literature related to regional lung health policy

The second phase of work will seek to answer the following, in addition to expanding on the research questions from phase one:

1. What factors influence lung health policy in Africa (or SSA)?
  a What international, economic or cultural factors influence regional policies, investments and commitments to lung health?
  b Are regional bodies influencing, in any way, member country policies, investments and commitments to lung health? For instance, have they required specific action from member countries to tackle lung health issues?
2. What investments are regional bodies currently making in lung health? What investments are needed by regional bodies to promote behaviours and environments which protect lung health?
3. What structures are in place to facilitate discussion and advocacy for lung health at regional level?
4. What is the role of evidence in addressing barriers to promoting and sustaining behaviours and environments that protect lung health at the regional level?

#### Search strategy

The review will be limited to peer-reviewed articles and grey literature published between 2008 and 2020. The following search terms will be applied: [Lung OR respiratory] AND [disease OR illness OR infection] AND [chronic OR acute] OR [TB OR tuberculosis OR chronic obstructive pulmonary disease OR COPD OR asthma OR acute respiratory infection* OR pneumonia OR severe acute respiratory syndrome OR respiratory syncytial virus OR RSV] AND [policy OR strategy OR program* OR advocacy] AND [policymak* OR practi* OR stakeholder*] AND [Africa] AND [East* OR South* OR West* OR Central*]. HINARI, JSTOR, Google Scholar and websites of organisations focusing on lung health policy, programmes and advocacy will be used to search for peer-reviewed and grey literature.

#### Inclusion criteria

Scientific articles will be eligible for inclusion if they focus on acute or chronic lung health policy, programme and strategy development, implementation or advocacy, including content and implementation gaps and challenges, and the role of evidence in acute or chronic lung health policy and programme development and implementation. Articles not focusing on lung health from the perspective of policy development and implementation and those in languages other than English will be excluded.

#### Screening

After removal of duplicates, abstracts of retrieved articles will be screened against the inclusion and exclusion criteria. Subsequently, remaining papers will be read in full and screened against the inclusion and exclusion criteria. Papers shall be shared between and reviewed by two reviewers using an Excel spreadsheet that, as in phase one, details any policies, programmes or interventions, date and aim, involved stakeholders and their interests, and policy and programme gaps, challenges and options. Papers retained by both reviewers after the first round of screening will then be reviewed by a third reviewer. Differences will be discussed between the three reviewers until consensus is reached.

#### Analysis

The reviewers will sort the selected papers by theme individually, and then come together to discuss any inconsistencies. Once consensus is reached, the researchers will generate a narrative synthesis based on their discussions. This will summarise emergent themes and patterns in the main findings of the studies to address each research question, including the factors influencing lung health policy in Africa, investments by regional bodies, regional decision-making and advocacy platforms, and the role of evidence. This will be used to develop a draft policy engagement strategy for the programme that provides evidence-based detail on regional lung health policies, actors, issues, structures, barriers and opportunities, guided by the Overseas Development Institute’s Rapid Outcome Mapping Approach [7].

### Phase 3: survey of IMPALA lung health experts and interview of key regional stakeholders

The third phase will generate empirical evidence to supplement the evidence from Phases 1 and 2 using an online qualitative survey and in-depth interviews. A synopsis of the findings from Phases 1 and 2, including a draft policy engagement strategy for the programme, will be shared with IMPALA partners and researchers, along with a survey designed to allow participants to confirm or suggest modifications to the emerging engagement strategy, including about key regional long health policies, policy priorities, gaps and implementation challenges. This will also ask IMPALA partners and researchers about their and other stakeholders’ involvement in regional policy development and implementation, advocacy and research, including their interests and power to influence change and key decision-making platforms for lung health. Key stakeholders with expertise in lung health policy in Africa identified from the survey will be interviewed to obtain additional insights on influencers of lung health policy in Africa to ensure the strategy is well-designed and directed. An interview guide will be used to conduct the interviews and its design will draw on the findings of the previous phases in order to elaborate upon themes identified and any evidence gaps.

#### Sampling

The survey will be administered to 5 research programme leads, 11 country leads, 5 PhD students and 5 post-doctoral research associates. Purposive sampling will be performed and interviews will be conducted with approximately 10–15 key stakeholders, including policy-makers, implementers and funders, with expertise in lung health policy in Africa.

#### Data collection and analysis

Survey Monkey will be used to gather the survey data, manage the survey dataset and conduct data analysis. Additional analysis may be conducted in Excel or NVivo as needed. Responses to open-ended survey questions will be post-coded to be transformed into quantitative data. Themes will be discussed between two researchers until consensus is reached. Descriptive analysis will be used to analyse the data by survey question.

Key informant interviews will be conducted by two researchers face-to-face or online via Bluejeans, GoToMeeting or Skype, dependent on the researchers and interviewees’ locations. The interviews will be semi-structured and designed to last approximately 45 minutes. The researchers will receive written consent as hard or soft copy as well as oral consent to record the interview. The interviews will be transcribed and coded by two researchers who will discuss emergent themes until consensus is met. Descriptive analysis will be used to analyse the interview findings, manually or using NVivo, as needed. This analysis will inform revisions to the IMPALA policy engagement strategy.

#### Ethics

Ethical approval will be sought from Liverpool School of Tropical Medicine and the National Health Science Research Committee in Malawi.

## Discussion

There have long been calls to extend the use of health policy analysis in low- and middle-income countries as literature has shown the importance of incorporating “*politics, process and power*” into studies involving health policy and systems [8–10]. This is because health policy analysis can meet health aims by showing why some health issues receive more attention than others, identifying barriers in implementation and identifying stakeholder positions on health issues, which can be used to develop strategies to advance reforms [11]. To inform IMPALA’s regional policy engagement, research uptake and accountability strategy, the regional lung health policy and stakeholder analysis seeks to generate an understanding of such essential issues within the landscape in which IMPALA is operating.

The methods outlined and the findings they yield are well positioned to achieve this objective. Each phase of the analysis, by design, builds on the foundation of the previous phase in order to contextualise findings. Specifically, the first phase will provide a general picture of regional support and investment in the various lung health illnesses by identifying key policies and actors as well as stakeholders’ relevant programmes and platforms. The second phase will then shed light on the drivers of the findings in Phase 1 by looking at the literature on different lung health issues. Finally, the third phase will bring findings from Phases 1 and 2 to life by explaining the unwritten and informal knowledge around lung health policy in Africa.

Beyond filling a major evidence gap on lung health policy in Africa, the analysis findings are critical if the research generated in IMPALA is to have influence beyond academia. Ultimately, the generated evidence will identify key lung health stakeholders, policies and platforms that IMPALA should leverage to maximise the policy impact of the multidisciplinary lung health research generated through the programme.

## Data Availability

Data sharing is not applicable to this article as no datasets were generated or analysed in the development of this protocol.

## Abbreviations

IMPALA: International Multidisciplinary Programme to Address Lung Health and TB in Africa
SSA: sub-Saharan Africa
TB: tuberculosis
